# Supplementary material for: Resolving the full spectrum of human genome variation using Linked-Reads
Source: Genome Res. 2019 Apr;29(4):635–45. doi: 10.1101/gr.234443.118 (PMC6442396; doi:10.1101/gr.234443.118)
Supplement: Supplemental Material [file supp_gr.234443.118_Supplemental_longranger-2.2.zip › longranger-2.2.tar.bz2!/lr2.2-public/lib/stan/cmdstan/stan/doc/api/html/mdivide__right__tri_8hpp_source.html]

Stan: src/stan/math/matrix/mdivide\_right\_tri.hpp Source File


|  |  |
| --- | --- |
|  | Stan  2.5.0  probability, sampling & optimization |


- Main Page
- Related Pages
- Namespaces
- Classes
- Files

- File List
- File Members

All Classes Namespaces Files Functions Variables Typedefs Enumerations Enumerator Friends Macros Pages

- src
- stan
- math
- matrix

mdivide\_right\_tri.hpp

Go to the documentation of this file.

1 #ifndef STAN\_\_MATH\_\_MATRIX\_\_MDIVIDE\_RIGHT\_TRI\_HPP

2 #define STAN\_\_MATH\_\_MATRIX\_\_MDIVIDE\_RIGHT\_TRI\_HPP

3

4 #include <stdexcept>

5 #include <boost/math/tools/promotion.hpp>

6 #include <stan/math/matrix/Eigen.hpp>

7 #include <stan/math/matrix/mdivide\_left\_tri.hpp>

8 #include <stan/math/matrix/transpose.hpp>

9 #include <stan/math/error\_handling/matrix/check\_multiplicable.hpp>

10 #include <stan/math/error\_handling/matrix/check\_square.hpp>

11

12 namespace stan {

13  namespace math {

14

24  template <int TriView, typename T1, typename T2,

25  int R1, int C1, int R2, int C2>

26  inline

27  Eigen::Matrix<typename boost::math::tools::promote\_args<T1,T2>::type,R1,C2>

28  mdivide\_right\_tri(const Eigen::Matrix<T1,R1,C1> &b,

29  const Eigen::Matrix<T2,R2,C2> &A) {

30  stan::math::check\_square("mdivide\_right\_tri(%1%)",A,"A",(double\*)0);

31  stan::math::check\_multiplicable("mdivide\_right\_tri(%1%)",b,"b",

32  A,"A",(double\*)0);

33  // FIXME: This is nice and general but requires some extra memory and copying.

34  if (TriView == Eigen::Lower) {

35  return transpose(mdivide\_left\_tri<Eigen::Upper>(transpose(A),transpose(b)));

36  }

37  else if (TriView == Eigen::Upper) {

38  return transpose(mdivide\_left\_tri<Eigen::Lower>(transpose(A),transpose(b)));

39  }

40  else {

41  throw std::domain\_error("triangular view must be Eigen::Lower or Eigen::Upper");

42  }

43  }

44

45  }

46 }

47 #endif

check\_multiplicable.hpp

transpose.hpp

stan::math::mdivide\_right\_tri

Eigen::Matrix< typename boost::math::tools::promote\_args< T1, T2 >::type, R1, C2 > mdivide\_right\_tri(const Eigen::Matrix< T1, R1, C1 > &b, const Eigen::Matrix< T2, R2, C2 > &A)

Returns the solution of the system Ax=b when A is triangular.

**Definition:** mdivide\_right\_tri.hpp:28

stan::math::check\_multiplicable

bool check\_multiplicable(const char \*function, const T1 &y1, const char \*name1, const T2 &y2, const char \*name2, T\_result \*result)

**Definition:** check\_multiplicable.hpp:15

stan::math::transpose

Eigen::Matrix< T, C, R > transpose(const Eigen::Matrix< T, R, C > &m)

**Definition:** transpose.hpp:12

mdivide\_left\_tri.hpp

Eigen.hpp

stan::math::check\_square

bool check\_square(const char \*function, const Eigen::Matrix< T\_y, Eigen::Dynamic, Eigen::Dynamic > &y, const char \*name, T\_result \*result)

Return true if the specified matrix is square.

**Definition:** check\_square.hpp:24

check\_square.hpp


---

[ Stan Home Page ]

*© 2011–2014,
Stan Development Team.*
